# Evaluating the ***In****Signia IFI27* expression assay for detecting viral respiratory infection compared to a traditional gene normalisation assay

**DOI:** 10.1038/s41598-025-04688-9

**Published:** 2025-07-01

**Authors:** Tiana M. Pelaia, Karan Kim, Nicole Lima, Claire Gibbs, Lee M. Smith, Sally Teoh, Ya Wang, Colin Denver, Elisa Mokany, Alison Todd, Sam Orde, Benjamin Tang, Anthony McLean, Maryam Shojaei

**Affiliations:** 1 Department of Intensive Care Medicine, Nepean Hospital, Penrith, NSW Australia; 2 Centre for Immunology and Allergy Research, The Westmead Institute for Medical Research, The University of Sydney, Westmead, NSW Australia; 3 SpeeDx Pty Ltd, Eveleigh, NSW Australia; 4 Faculty of Medicine and Health, Sydney Medical School Nepean, Nepean Hospital, University of Sydney, Penrith, NSW Australia

**Keywords:** Gene expression analysis, Transcription, Diagnostic markers

## Abstract

Host gene expression is crucial for understanding disease progression and developing diagnostic biomarkers. Previously, we identified a novel immune biomarker *IFI27*, validated with routine RT-qPCR methods employed in a research setting, that discriminates between influenza and bacteria in patients with suspected respiratory infection. This study aimed to assess the ***In****Signia* method, which employs a novel gene normalization technique to yield a variable transcript analysis (VITA) index. The VITA index measures gene expression relative to a non-transcribed region of DNA, such that it is independent of sample quality or quantity. We compared *IFI27* gene expression measured by the ***In****Signia* assay to that of the research assay in blood samples collected from patients with respiratory diseases and SARS-CoV-2 vaccinated individuals. The study found a strong correlation and acceptable agreement between traditional ΔCq methods and ***In****Signia* for *IFI27* levels in the higher range (log(ΔCq)_Research_ > 1), but not for *IFI27* expression levels below this range, likely due to the different normalization strategies. Notably the ***In****Signia* assay was more sensitive in detecting viral infection among hospital patients. These findings suggest that the ***In****Signia* assay, which supports high throughput workflows, may be used for the rapid detection of viral infection in patients with respiratory symptoms.

**Supplementary Information:**

The online version contains supplementary material available at 10.1038/s41598-025-04688-9.

## Introduction

Gene expression is a key measure of how biological systems are regulated at a molecular level. Studying transcriptional activity has not only heightened our understanding of the mechanisms underpinning disease progression but has led to the development of promising RNA biomarkers that form the basis of diagnostic and prognostic tests^1,2^. Gene expression platforms that measure transcript abundance are swiftly evolving, and include quantitative PCR (qPCR), microarrays, and RNA sequencing (RNASeq). While these assays are indispensable players in gene expression studies, it is the rapidity, accessibility and cost-effectiveness of qPCR that makes it the most clinically feasible option in the early detection of disease^3,4^.

Notably, measurement of messenger RNA (mRNA) in blood samples using reverse transcription qPCR (RT-qPCR) has garnered considerable attention in the past decade, as there is potential to wield it as a diagnostic tool for cancers, bacterial infections and sepsis^5–9^. For instance, the host mRNA *IFI27* is a blood immune biomarker for early viral infection^10–15^. *IFI27* has offered a fresh outlook on managing patients with symptoms that manifest in both bacterial and viral infections and has been validated in prospective studies using a well-established research laboratory workflow that involves manual RNA extraction followed by RT-qPCR amplification using a probe-based TaqMan assay, with housekeeping genes for normalization^10,11^.

However, the emergence of the SARS-CoV-2 pandemic highlights the need to enhance conventional RT-qPCR detection tools to help clinicians make faster, more informed decisions^16^. The selection of nucleic acid extraction method, RT-qPCR assay type, amplification platform and normalisation strategy are amongst several important considerations required to effectively deliver biomarker research to the bedside^17^. By optimizing these factors, the ability to obtain early and accurate differential diagnoses can be significantly improved, leading to better patient outcomes.

The ***In****Signia* (SpeeDx Pty Ltd) assay utilizes a novel technique to quantify gene expression, whereby the target gene of interest (GOI) and its associated transcripts are normalized to a non-expressed region of DNA (NED)^18,19^. Expression levels are calculated as a variable transcript analysis (VITA) index using the formula [2^ (C_*q*_ NED - C_*q*_ *GOI*)] / TR, which is an adaptation of the traditional ΔCq method, whereby TR is the theoretical ratio of the number of copies of DNA of the GOI comparative to the NED. The VITA index yields a numerical value reflective of the relative amount of RNA present in the sample per DNA copy. As opposed to traditional normalisation methods, the ***In****Signia* technique relies on the concurrent extraction and detection of both RNA and DNA nucleic acid species, which eliminates issues with potential DNA contamination within RNA preparations. It utilises an organism-specific, stable, tissue and disease-independent, DNA-only region for normalisation of RNA levels from the GOI which itself is detected in both DNA and RNA form to provide greater accuracy when normalising against DNA. All this enables internal gene expression normalisation from one sample in a single reaction^18,19^.The method is probe-independent, however, within this study, ***In****Signia* employs ***Plex****PCR* technology for superior multiplexing and high-throughput capacity from an automated workflow that is much more capable of meeting unprecedented testing demands and delivering results rapidly^20^. The ***In****Signia* method has been developed for the assessment of viability and rapid antibiotic susceptibility testing of bacteria^21^, yet its clinical utility for the normalisation of human genes is currently unknown.

This study aims to compare the research-based assay and the ***In****Signia* assay by assessing (i) quantitative *IFI27* measurements and (ii) performance in detecting viral infection in respiratory patients. By conducting this comparison, we seek to determine the accuracy of the novel normalisation methods used by ***In****Signia* and its applicability in clinical settings.

## Materials/methods

### Ethics statements

The study was approved at Western Sydney Local Health District (HREC Reference: 2020/ETH00886), Research Governance at Westmead Institute for Medical Research, and Nepean Blue Mountains Local Health District (HREC Reference: 2019/ETH01485 and 2021/ETH00222). Written informed consent was obtained from all study participants. All methods were performed in accordance with institutional guidelines and regulations.

### Study design

A total of 141 participants are included in this study, with 95 confirmed cases of viral infection, 23 cases of non-viral infection and 23 healthy individuals. To capture a full spectrum of *IFI27* expression profiles, samples were prospectively collected from three cohorts drawn from an array of settings to enhance the coverage of a heterogeneous participant population. Participants were recruited from studies validating *IFI27* in respiratory viral infections and included hospital patients from respiratory wards with well-defined phenotypes (viral infection, bacterial infection, and non-infectious respiratory illnesses). As *IFI27* is implicated in the innate immune response, 23 healthy volunteers prior to or after receiving SARS-CoV-2 vaccinations were also included. Demographic and clinical characteristics of study cohorts are summarized in Table 1. Moreover, detailed information on all cohorts (inclusion criteria, recruitment process, sample collection and follow-up) is presented below.

### Study cohorts

*Cohorts 1 and 2*: Study participants were individuals with suspected respiratory infections during 2014–2022. Subjects with recent (within the prior 14 days) vaccination history, infection/under antimicrobial medication, and subjects under immunosuppressive drugs were not included in the study. Eligibility for the study occurred upon the reporting of suspected flu-like symptoms (e.g., fever, sore throat, cough). Individuals were considered viral patients (cohort 1) if positive for a virus (Influenza A, Influenza B, Parainfluenza, RSV, Adenovirus, Enterovirus, Rhinovirus, or Metapneumovirus) via virological testing on respiratory samples (nasal, throat, swab, sputum, or bronchoalveolar lavage). Individuals were considered bacterial patients (cohort 2) if positive for bacteria cultured from nasopharyngeal, oropharyngeal, or midtubinate; sputum or saliva; bronchoalveolar lavage or endotracheal tube; or other specimens (typically blood and urine), or if a bacterial infection was suspected by their consulting physician. Blood samples were collected at enrolment on admission or in the subsequent 28-day follow-up period.

*Cohort 3*: Study participants were individuals who were 18 years or older undergoing voluntary SARS-CoV-2 vaccination. While only one sample was randomly selected for analysis for the purposes of this study, four blood samples in total were collected from each participant; sample 1 was taken immediately before the first vaccination injection, sample 2 and sample 3 were taken 2–5 days after the first and second vaccination injections respectively, and sample 4 was taken 14 days post-second vaccination injection.

**Table 1:** Overview of cohorts included in the study.

	Cohort 1 (*n* = 95)	Cohort 2 (*n* = 23)	Cohort 3 (*n* = 23)
Description	Viral patients	Non-viral patients	Healthy volunteers
Age^a^	43.16 (16.14)	56.26 (14.91)	43.63 (16.04)
Sex (male/female)	45/50	12/11	7/16
Locations of recruitment	Czech Republic (3) Australia (92)	Australia (23)	Australia (23)
Species identified or diagnosis	SARS-CoV-2 (82) Rhino/enterovirus (5) Influenza A (2) Adenovirus (1) Influenza B (1) SARS-CoV-2 + bacterial co-infection (4)	Bacterial infection (confirmed or suspected) e.g. S. aureus, S. pneumoniae, tonsilitis, CAP (4) Non-infectious respiratory illness e.g. exacerbation of asthma, COPD, pleuritic pain, rhinorrhoea (19)	None

^a^Age presented as means (SD).

CAP = Community acquired pneumonia; COPD = Chronic obstructive pulmonary disease.

### Sample collection

Whole blood (2.5 ml) was collected into PAXgene Blood RNA tubes (Qiagen) from 141 participants according to the manufacturer’s instructions. Of these, 118 blood samples were collected from study participants 0–18 days after presentation to the hospital with “flu-like” symptoms, with one sample collected per patient. In the case of the 23 healthy volunteers, PAXgene blood sample collection was performed before and after the administration of SARS-CoV-2 vaccination, but only one sample per participant was randomly selected for analysis. All blood samples were stored at − 80 °C as aliquots from the original sample until they were tested on both assays. A pilot study to understand the effects of blood collection method by comparing PAXgene and EDTA blood samples is detailed in the supplementary methods.

### Research assay

Total RNA was isolated and purified from all blood samples using the PAXgene Blood RNA kit (QIAGEN). The concentration of the isolated RNA was evaluated using NanoDrop (Thermo Fisher) and stored at − 80 °C for long-term storage. Complementary DNA (cDNA) was synthesized from the 500ng of isolated RNA using a qScript cDNA SuperMix (Quantabio) according to the manufacturer’s guidelines. Singleplex RT-qPCR was performed in a total reaction volume of 10µL per reaction containing 4µL of synthesised cDNA or water (negative) as a template, and 5 µl of 2× TaqMan gene expression Master Mix (Thermo Fisher Scientific). Primer sets were used, targeting *IFI27* (Assay ID: Hs01086370_m1-FAM) and *GAPDH* (Assay ID: Hs99999905_m1-VIC). qPCR was performed in a 384-well plate on the CFX384 (Bio-Rad) with the following thermal cycler programme: Polymerase activation, 10 min at 95 °C; denaturation, 15 s at 95 °C; annealing and extension, 60 s at 60 °C. The denaturation and annealing steps were repeated for 40 cycles. The quantification cycle (C_*q*_) values from the RT-qPCR were measured through fluorescent dyes targeting probes FAM and VIC. The ΔC_*q*_ method was used to calculate the fold change in gene expression, utilizing *GAPDH* as a housekeeping gene. The RT-qPCR amplification efficiencies for the *IFI27* and *GAPDH* consistently ranged from 95 to 98%. The methods were designed in accordance with published recommendations for qPCR^22^.

### ***In****Signia* assay

Samples were extracted using the MagNA Pure 96 DNA and Viral NA small volume kit (Roche) using the Pathogen Universal 200 protocol. Blood samples were diluted 1:10 in phosphate buffered saline (PBS) before extraction. An internal control consisting of PlexPCR internal control RNA cells (SpeeDx Pty Ltd) diluted 1:100 in PBS, was added (20 µL per sample) on-board the MagNA Pure 96 (Roche). A sample input volume of 200 µL and an elution volume of 50 µL was used. The ***In****Signia IFI27* assay was prepared using the PlexPCR master mix (SpeeDx Pty Ltd). The master mix was a total of 15 uL, consisting of 10 µL Plex Master Mix (2X), 0.4 µL reverse transcriptase (50X), 0.4 µL RNase inhibitor (50X), 1 µL ***In****Signia IFI27* assay mix (20X), and 3.2 µL nuclease-free water. Addition of nucleic acid extracts (5 µL) created a 20 uL reaction volume. ***In****Signia* amplification and detection was performed in a 96-well plate on the LightCycler 480 II instrument (Roche) using the following cycling parameters: 48 °C for 10 min (reverse transcriptase), 95 °C for 2 min (polymerase activation), 10 cycles of 95 °C for 5 s and 61 °C (− 0.5 °C per cycle) for 30 s (touchdown cycling), 40 cycles of 95 °C for 5 s and 52 °C for 50 s (quantification cycling), and 40 °C for 30 s (cooling). Detection occurred in four channels: (i) 465–510 nm (FAM) for detection of the GOI (*IFI27* DNA and RNA), (ii) 533–610 nm (Texas Red) for detection of normalizing non-expressed DNA (NED), (iii) 533–580 nm (JOE) for detection of the internal control sequence, and (iv) 618–660 nm (Cy5) for detection of the RNA integrity control. Data was analysed on the LightCycler 480 software using Abs Quant/Second derivative max method to obtain the C_*q.*_ The internal control was used to monitor sample extraction efficiency and PCR inhibition (expected ≤ 22 *C*_*q*_) and the RNA integrity control was used to assess the quality of RNA (< 26 *C*_*q*_). Transcription of the *IFI27* gene was measured using the VITA method, which is a ratio of the number of copies of a gene and its associated transcripts to those of a non-transcribed region of DNA. The formula [2^ (C_*q*_ NED - C_*q*_*IFI27*)] / 0.5 was used to calculate the VITA index, where 0.5 is the TR, calibrating for the number of copies present in the *IFI27* gene (one copy) compared to the non-transcribed DNA (two copies). As an illustrative example, a C_*q*_ NED of 18.5 and a C_*q*_ *IFI27* of 16.2 would result in a VITA index of 9.8. The VITA index calculation also considers the qPCR amplification efficiency, which are estimated at 102.6% and 97.2%, for NED and *IFI27* detection respectively.

### Statistical analysis

*Comparison of quantitative IFI27 measurements*: The raw *IFI27* ΔC_*q*_ values derived from the research assay were analysed with the nonparametric Kruskal-Wallis test to assess differences between the viral patients (cohort 1), non-viral patients (cohort 2) and healthy volunteers (cohort 3). This analysis was repeated with the raw VITA Index values obtained from the ***In****Signia* assay. A *p* value of < 0.05 was considered statistically significant. *IFI27* measurements from the research and the ***In****Signia* assays were then log_10_ transformed and compared using correlation and Bland–Altman analyses. As the *IFI27* values obtained from two assays exhibited different linear trends across the dataset, we performed a segmental correlation analysis to better understand these relationships. Bland-Altman analysis was conducted specifically on the segments that demonstrated a significant linear relationship between the two assays to assess their agreement. First, the data within the segment was randomly divided 2:1 into a training set and a test set using random number generation. A linear regression was conducted on the training set to derive a predictive equation, allowing us to estimate values for the ***In****Signia* assay (*y*) based on measurements from the research assay (*x*). This was necessary to align the different units of measurement used by the two assays. The regression equation was then applied to the test set to generate predicted values for the ***In****Signia* assay. Finally, Bland-Altman analysis was conducted on the test set to evaluate the agreement between the ***In****Signia* VITA index values predicted from the research assay, and the actual ***In****Signia* VITA index *IFI27* measurements. This methodology provides a robust verification strategy to assess the performance of the linear regression model on unseen data, facilitating a thorough evaluation of the agreement between the two assays.

*Diagnostic performance*: To compare the performance of both assays in detecting viral infection, the dataset from cohorts 1 and 2 was randomly divided 2:1 into a training set and a test set using random number generation. The optimal *IFI27* cut-off levels were calculated from the training set using a receiver operator characteristic (ROC) curve analysis, with reference to the results from the hospitals’ respiratory pathogen panel as the “gold standard”. Three samples were excluded on the basis that the time between hospital presentation and sample collection was > 7 days, to align with the objective of investigating *IFI27* as an early marker for viral infection in a fast-paced medical context. Using these cut-off values, the sensitivities and specificities of the research and ***In****Signia* assays were calculated in the test set. The training-test approach ensures that the optimal *IFI27* cut-off levels are derived from independent data, reducing the risk of overfitting and allowing for more accurate validations of the assays’ sensitivities and specificities in a new dataset. All statistical analyses were performed using GraphPad Prism version 10.0.2.

## Results

### Comparison of quantitative *IFI27* measurements

**Fig. 1:**
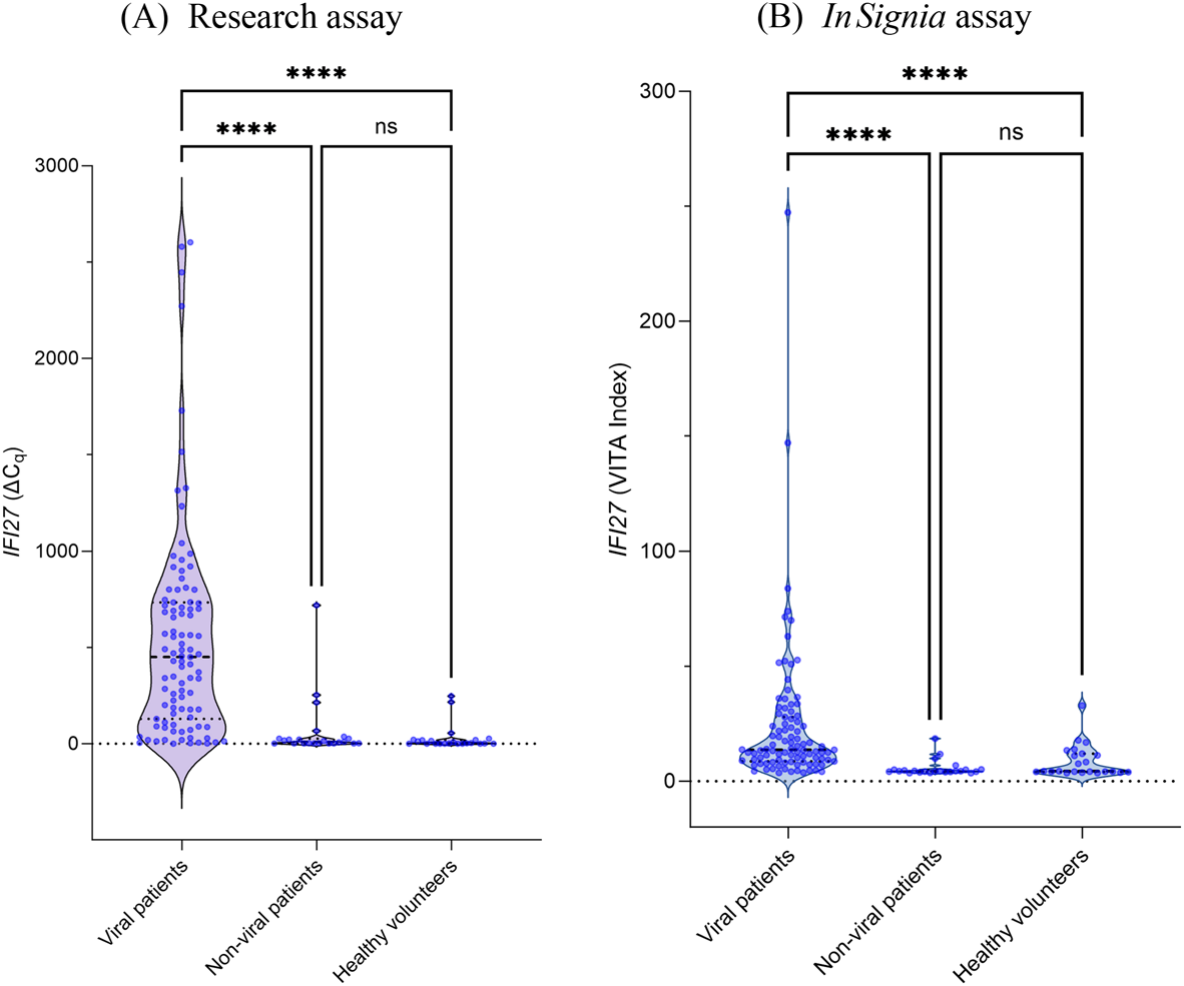
Violin plots of the *IFI27* expression profiles from the (**A**) research and (**B**) ***In****Signia* assays, grouped by viral patients (*n* = 95), non-viral patients (*n* = 23), and healthy volunteers undergoing SARS-CoV-2 vaccination (*n* = 23). Horizontal dashed lines represent means ± standard error of the mean (SEM).

**Fig. 2:**
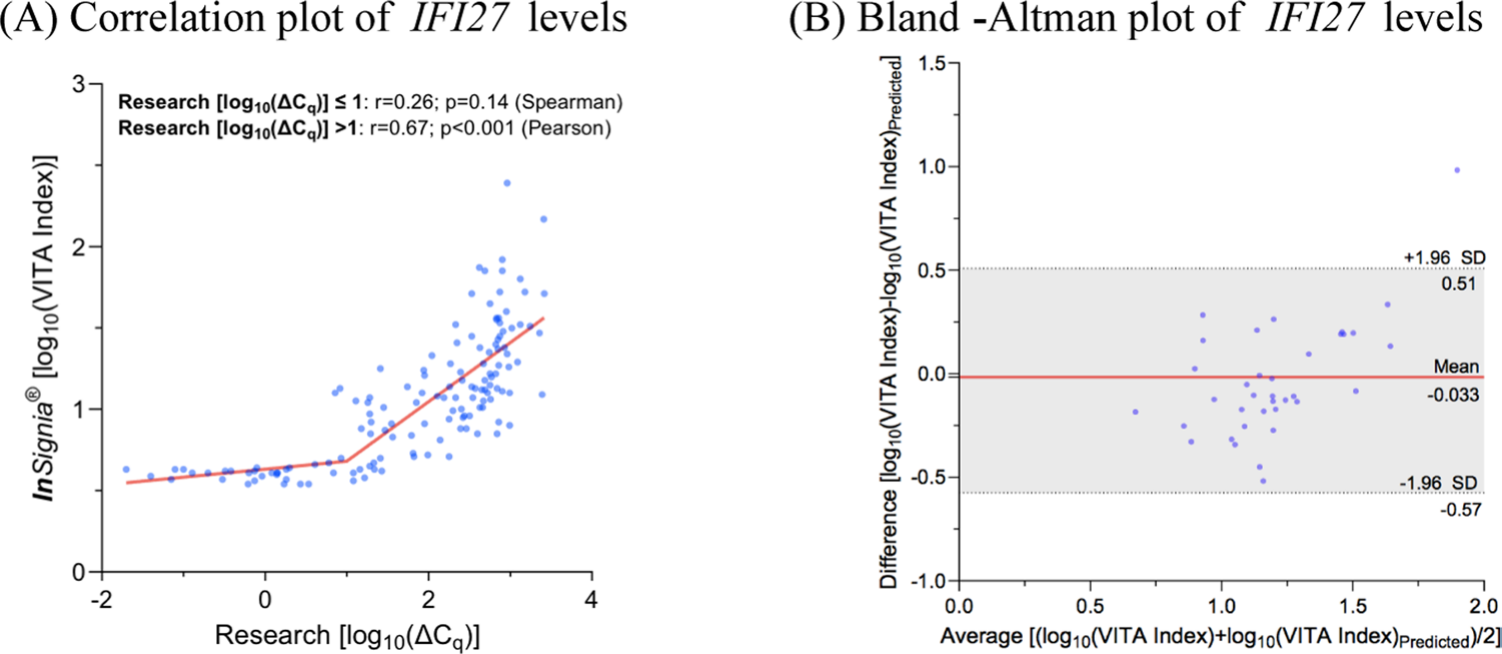
(**A**) Segmental correlation between *IFI27* measurements using the research assay and the ***In****Signia* assay (*n* = 141) and (**B**) Bland-Altman plot of *IFI27* measurements derived from a test set of the research assay (in which the measurements have been interpolated into predicted values) and the ***In****Signia* assay (*n* = 36), for measurements where log(ΔC_*q*_)_Research_>1 (i.e. the second segment). Grey shaded areas represent 95% confidence limits.

The *IFI27* expression profiles of blood samples performed with the research and ***In****Signia* workflows are presented in Fig. 1, illustrating the raw ΔC_*q*_ and VITA index values amongst the three study cohorts. Both assays revealed a significant upregulation of *IFI27* in the viral cohort compared to the non-viral patients (*p* < 0.001) and healthy volunteers (*p* < 0.001). There were no differences in *IFI27* levels between the non-viral and healthy volunteer cohorts, for both the research assay (*p* = 0.1096) and the ***In****Signia* (*p* = 0.4902). The data these cohorts were log_10_ transformed for analyses comparing the two assays as shown in Fig. 2. The correlation plot in Fig. 2A reveals two distinct linear segments, intersecting at log(ΔC_*q*_)_Research_=1. Based on sample size and distribution characteristics, the Spearman’s test was employed for the first segment, with raw ΔCq values ranging from 0.02 to 8.48 for the research assay, which revealed a weak and non-significant correlation (*r* = 0.26, *p* = 0.1446). For the second segment with raw ΔCq values ranging from 12.00 to 2602.34 for the research assay, the Pearson’s test was appropriate and revealed a clear, positive linear correlation between results from the research assay and ***In****Signia* assay (*r* = 0.67, *p* < 0.001). We then randomly divided the measurements from the second segment 2:1 into a training set (*n* = 73) and test set (*n* = 36). A Bland-Altman analysis was conducted on the test set using a regression equation obtained from the training set: log(VITA Index)_***In****Signia*_ = 0.3692*log(ΔC_*q*_)_Research_ + 0.3139. This equation was used to predict log(VITA Index) values based on measurements from the research assay. The mean difference between the predicted log(VITA Index) and observed log(VITA Index) was − 0.033 (SD 0.2765), with limits of agreement ranging from − 0.575 to 0.509. Out of the 36 paired samples in the test set, it was found that 24 had a difference of < 0.25 log(VITA Index), 34 had a difference of < 0.5 log(VITA Index), and 36 had a difference of < 1 log(VITA Index). Figure 1B illustrates that the difference between predicted and observed *IFI27* levels, which tends to increase at higher expression levels. The results from a pilot study comparing the log(VITA Index) values of samples collected from PAXgene blood RNA tubes and EDTA tubes is presented in the supplementary material (Fig S1), and demonstrates a strong, significant correlation between sample collection methods (*r* = 0.944, *p* < 0.001).

### Comparison of performance in detecting viral infection in respiratory patients

**Fig. 3:**
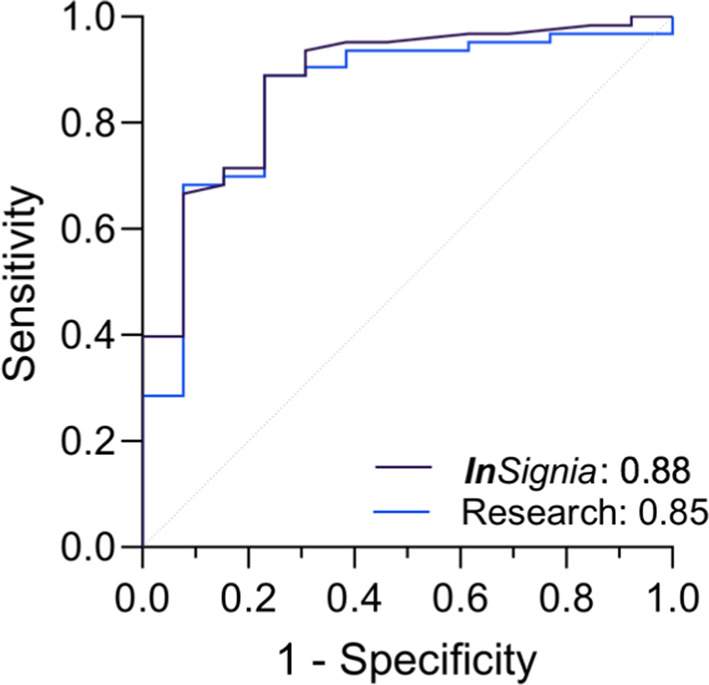
Area-under-the-curve of Receiver-Operator-Characteristics curve (AUROC) analysis of blood *IFI27* gene expression levels in detecting viral infection in a training set of cohorts 1–2 (*n* = 76) using the research assay and the ***In****Signia* assay.

We divided the viral and non-viral cohorts 2:1 so that the diagnostic threshold was optimized in the training set (*n* = 76), which was then used to calculate the sensitivity and specificity on the test set (*n* = 38). We found that a threshold of ΔC_*q*_ = 74 for the research assay and VITA Index = 6.9 for the ***In****Signia* assay provided the most optimal level of *IFI27* expression to differentiate between viral and non-viral infections. As shown in Fig. 3, the AUROC for the research-based assay was 0.85 (95% CI = 0.7357 to 0.9679) and the AUROC for the ***In****Signia* assay was 0.88 (95% CI = 0.7734 to 0.98). Using this cut-off value in the test set, the sensitivity of using *IFI27* as a predictor of viral infection was 76% with specificity of 100%for the research assay, and a sensitivity of 93% with specificity of 100% for the ***In****Signia* assay. The derivation of these values is presented as contingency matrices in Table 2. A comparison of the diagnostic performance between the two assays, including positive predictive values, negative predictive values, and accuracy, is presented in Table 3.

**Table 2:** Contingency matrices for the test set of the (A) research assay and (B) ***In****Signia* assay, defining the true positive, true negative, false positive, and false negative rates for detecting viral infection, with reference to the clinical respiratory pathogen panel as the ‘gold standard’ (*n* = 38).

(A)	Respiratory pathogen panel			(B)	Respiratory pathogen panel		
Research assay	Viral	Non-viral	Total	***In*** *Signia* assay	Viral	Non-viral	Total
Viral	22 (TP)	0 (FP)	22	Viral	27 (TP)	0 (FP)	27
Non-viral	7 (FN)	9 (TN)	16	Non-viral	2 (FN)	9 (TN)	11
Total	29	9	**38**	Total	29	9	**38**

TP = True Positive; FP = False Positive; FN = False Negative; TN = True Negative

**Table 3:** Comparative diagnostic performance of the research assay and ***In****Signia* assay in detecting viral infection (*n* = 38).

Metric	Research assay	***In****Signia* assay
Sensitivity [%, (95% CI)]	75.86 (56.46–89.70)	93.10 (77.23–99.15)
Specificity [%, (95% CI)]	100 (66.37–100)	100 (66.37–100)
Positive Predictive Value [%, (95% CI)]	100 (84.56–100)	100 (87.23–100)
Negative Predictive Value [%, (95% CI)]	56.25 (40.28–71.02)	81.82 (54.16–94.49)
Accuracy [%, (95% CI)]	81.58 (65.67–92.26)	94.74 (82.25–99.36)

## Discussion

This study validated the ***In****Signia* assay by comparing it with the well-established research assay for RT-qPCR detection of *IFI27*, a robust biomarker for the onset of viral illness. Across diverse cohorts including individuals with viral and non-viral respiratory conditions, as well as healthy participants, blood *IFI27* levels exhibited significant correlation and acceptable agreement between the two assays, but only for a higher range of values (log(ΔC_*q*_)_Research_>1). Both assays reliably detect viral infections in hospitalized patients, with the ***In****Signia* assay being more sensitive than research assay. Taken together, the results underscore the potential utility of the ***In****Signia* for gene normalisation in a research setting, but with also great promise in a clinical setting to discriminate viral from non-viral respiratory conditions.

As a host innate immune response gene, *IFI27* expression has been measured using RT-qPCR methods across various sources, including cell lines^23,24^, tissues in mouse models^25,26^, as well as in human blood^10–15,27^. The gold standard methods of qPCR using TaqMan probes and SYBR Green dye have been widely adopted for the measurement of *IFI27* gene expression due to their established accuracy and reliability in RT-qPCR analysis^28,29^. Of significance is the increasing application of these methods in studying elevated *IFI27* levels in the blood of patients infected with viruses like SARS-CoV-2^11,12^, influenza^10^, RSV^13^, and HIV^14,15^.

In this context, *IFI27* has been thoroughly validated as a blood biomarker for viral infections using RNA only as template and qPCR utilising TaqMan probes, with the diagnostic threshold of ΔC_*q*_ = 74 obtained in this study aligning with prior research findings^10^. Building upon this validation, the study introduces an innovative approach with the ***In****Signia* assay, which offers a streamlined and automated workflow for rapid nucleic acid extraction and gene expression quantification with normalisation against an invariable DNA sequence. It is a highly multiplexed reaction, with the incorporation of controls for sample extraction and adequacy, further increasing its commercial readiness without significant change to cost per reaction. In comparison to the research assay, the novel VITA index method demonstrates a strong correlation coefficient of 0.67 for samples with high *IFI27* expression values. However, at lower expression levels the correlation was lost, which may result from the different normalisation strategies. The research assay normalises to GAPDH mRNA, a housekeeping gene that can vary in expression levels, whilst the ***In****Signia* assay normalises to a sequence of DNA never present in RNA form and that does not vary in concentration regardless of sample type or disease state. This difference in normalization may lead to more consistent *IFI27* readings from the ***In****Signia* assay, particularly in non-viral conditions where *IFI27* is not expected to be upregulated. Moreover, the VITA index formula utilises an exponential term, which amplifies the sensitivity of the index to minor changes in C_q_ values in a manner that may not correlate linearly with the research assay at lower expression levels.

For the higher range of *IFI27* levels, the ***In****Signia* assay displayed on average a 0.033 log(VITA Index) lower value than those interpolated from the research assay. Simultaneously, the Bland-Altman plot exhibited more pronounced discrepancy between the values obtained from both assays as *IFI27* expression increases. This is once again likely due to the VITA index formula’s utilisation of an exponential term, in which minor changes in *C*_*q*_ translate into significant changes in the final VITA Index. Despite these differences, the ***In****Signia* assay outperforms the research assay in discerning viral from non-viral illness, when a diagnostic threshold of VITA Index = 6.9 is applied. In addition to differing normalization strategies, the ***In****Signia* assay features a refined workflow, including automated total nucleic acid (TNA) extraction, in contrast to the manual extraction used in the research assay. Furthermore, the ***In****Signia* assay integrates the reverse transcription and qPCR steps into a single reaction, whereas the research assay employs a two-step protocol which may introduce potential errors and biases that compromise sensitivity. Collectively, these methodological advantages could enhance the performance of the ***In****Signia* assay, thus presenting a valuable new avenue for investigating *IFI27* expression, where further improvements to its sensitivity in the lower range could provide an enhanced method for gene expression analysis.

To the best of our knowledge, this is the first study to evaluate the performance of the ***In****Signia* technology against conventional RT-qPCR methods in measuring gene expression levels in human samples. Our study’s strengths lie in the innovative approach to normalizing gene expression using ***In****Signia* technology. Normalisation is a crucial part of RT-qPCR studies to assess quantification efficiency and account for variability among samples. Typically, a reference gene is used alongside the gene of interest, where a key tenet is ensuring that the reference gene is stably expressed across samples^30–33^. This has so far proven to be a challenge, as the expression of commonly chosen reference genes can demonstrate considerable variability, including GAPDH^30,31,33^, in which mRNA levels are not always constant in whole blood^34^, as well as in human T-cells and PBMCs under viral stimulation^35^. The ***In****Signia* VITA index aims to circumvent this limitation by measuring transcription independently of sample type, quality or quantity, through the ratio of the copy number of informative gene and transcripts, compared to the stable NED region. Further research is warranted to assess the clinical significance of measuring *IFI27* in this manner, especially considering the observed gradient of *IFI27* levels in this study. Understanding the implications of these measurements in the context of a patient’s clinical status is crucial for advancing the utility of this diagnostic tool. A wide variety of viral and bacterial infections, as well as non-infectious respiratory conditions, present with common clinical manifestations where signs and symptoms overlap significantly, serving as a barrier to an accurate diagnosis^36–39^. This diagnostic uncertainty may lead to inappropriate treatments, including unnecessary antibiotic use^40,41^, and cause significant delays, particularly when relying on traditional diagnostic methods such as blood cultures^42^. The ***In****Signia* assay workflow has shown promise in addressing these challenges by rapidly (within 3 h of blood collection) distinguishing viral infections from non-infectious conditions and bacterial infections. This capability not only expedites the diagnostic process but also has profound implications for optimizing patient management strategies. Importantly, the ***In****Signia* assay demonstrates versatility in addressing the unmet need for rapid diagnostics that extend beyond viral infections, where *IFI27* can be replaced or complemented with additional biomarkers to effectively diagnose a range of conditions requiring urgent intervention.

Limitations of this study include the small sample size, particularly when dividing between training and test sets. Cohort 1 is enriched with samples from SARS-CoV-2 patients, potentially limiting the generalizability of results to other viruses. Additionally, there is a need for a more balanced representation of viral and non-viral patient samples to further verify the sensitivity and specificity of both assays, as well as a need to better understand the co-infected sample expression profiles. Furthermore, the use of PAXgene tubes for preservation of RNA once the sample was collected could have affected the results comparatively to expected collection devices used in clinical practice. To better mirror the urgent setting of a hospital environment where rapid diagnosis is crucial, further research is necessary to accurately simulate these conditions, utilizing already established protocols and resources. Preliminary findings from a pilot study suggest that measuring *IFI27* in blood from EDTA tubes within 2 h of collection may serve as a practical alternative to the PAXgene tubes used in this study (Fig S1). Moreover, the ***In****Signia* assay’s compatibility with microvolume samples (20 µL) allows for optimization with other readily accessible and less invasive sample types, broadening its potential applications in urgent clinical settings. This includes capillary blood sampling via finger sticks, nasopharyngeal samples for respiratory infections, and urine for bacterial testing as explored previously^21^.

## Conclusion

By employing a well-validated research assay utilising TaqMan as a point of comparison, we show that ***In****Signia* assay is a useful method for measuring *IFI27* mRNA expression, especially in relation to newly diagnosed viral cases where it has a higher sensitivity. The novel normalisation methods applied in ***In****Signia* demonstrate the potential to provide more accurate measures of gene expression compared traditional methods that use variable housekeeping genes.

## Electronic supplementary material

Below is the link to the electronic supplementary material.

Supplementary Material 1

## Data Availability

The datasets used and analysed during the current study are available from the corresponding author on reasonable request.
