# Supplementary material for: Evaluating the InSignia IFI27 expression assay for detecting viral respiratory infection compared to a traditional gene normalisation assay
Source: Sci Rep. 2025 Jul 1;15:21481. doi: 10.1038/s41598-025-04688-9 (PMC12216230; doi:10.1038/s41598-025-04688-9)
Supplement: Supplementary file 1 — Supplementary Material 1 [file 41598_2025_4688_MOESM1_ESM.docx]

**Supplementary methods**

**PAXgene vs EDTA sample collection pilot study:** Study participants were individuals with suspected respiratory infection, with samples collected on the day of presentation to the hospital upon reporting of suspected flu-like symptoms (e.g., fever, sore throat, cough) with samples collected on the day of presentation. Two blood samples were collected from each of the 19 patients that were recruited; one sample was collected in a PAXgene blood RNA tube for the immediate stabilization of intracellular RNA, and the other sample was collected in an EDTA tube (BD Vacutainer), which is readily available in hospitals and significantly less costly. Both samples were subjected to the ***In****Signia* assay workflow within 2 hours of collection as described in the methods. The corresponding VITA index values from the paired PAXgene and EDTA blood samples were log_10_ transformed and compared using Pearson’s correlation analysis.

**Supplementary Figure 1**


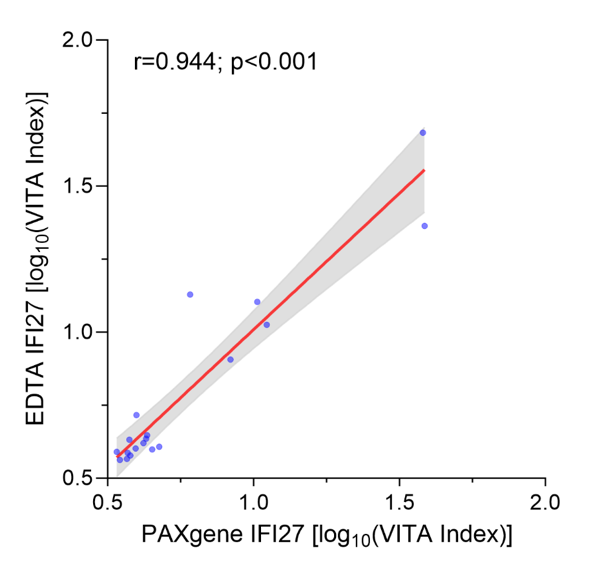


**Figure S1** Pearson’s correlation between *IFI27* measurements using the ***In****Signia* assay on paired PAXgene and EDTA blood samples 2 hours post collection (*n*=19). Grey shaded areas represent 95% confidence limits.
